# The Caspase-1/IL-18 Axis of the Inflammasome in Tumor Cells: A Modulator of the Th1/Tc1 Response of Tumor-Infiltrating T Lymphocytes in Colorectal Cancer

**DOI:** 10.3390/cancers13020189

**Published:** 2021-01-07

**Authors:** Linda Bilonda Mutala, Cécile Deleine, Matilde Karakachoff, Delphine Dansette, Kathleen Ducoin, Romain Oger, Olivia Rousseau, Juliette Podevin, Emilie Duchalais, Pierre Fourquier, Wassila El Alami Thomas, Pierre-Antoine Gourraud, Jaafar Bennouna, Camille Brochier, Nadine Gervois, Céline Bossard, Anne Jarry

**Affiliations:** 1 Institut Roche, 92100 Boulogne-Billancourt, France; linda.bilonda@univ-nantes.fr (L.B.M.); camille.brochier@roche.com (C.B.); 2 Inserm, CRCINA, Université de Nantes, 44000 Nantes, France; cecile.deleine@univ-nantes.fr (C.D.); kathleen.ducoin@etu.univ-nantes.fr (K.D.); romain.oger@univ-nantes.fr (R.O.); nadine.gervois@univ-nantes.fr (N.G.); celine.bossard@chu-nantes.fr (C.B.); 3 LabEx IGO, Université de Nantes, 44000 Nantes, France; 4 Clinique des Données, CHU de Nantes, INSERM, CIC 1413, 44093 Nantes, France; matilde.karakachoff@chu-nantes.fr (M.K.); Olivia.Rousseau@univ-nantes.fr (O.R.); pierre-antoine.gourraud@univ-nantes.fr (P.-A.G.); 5 Pathology Department, CHU Nantes, 44093 Nantes, France; delphine.dansette@chd-vendee.fr; 6 Digestive Surgery Department and IMAD, CHU Nantes, 44093 Nantes, France; juliette.podevin@chu-nantes.fr (J.P.); Emilie.DASSONNEVILLE@chu-nantes.fr (E.D.); 7 Digestive Surgery Department, Hôpital Privé du Confluent, 44200 Nantes, France; dr.fourquier@groupeconfluent.fr; 8 Institut d’Histopathologie, 44104 Nantes, France; welalami@ihp-pathologie.fr; 9 Digestive Oncology Department and IMAD, CHU, 44093 Nantes, France; jaafar.bennouna@chu-nantes.fr

**Keywords:** colorectal cancer, inflammasome, caspase-1/IL-18 axis, tumor-infiltrating T lymphocytes (TILs), Th1/Tc1 (IFNγ) response, ex vivo explant culture

## Abstract

**Simple Summary:**

The evolution of colorectal cancer (CRC) is influenced by complex interactions between tumor cells and tumor-infiltrating lymphocytes (TILs). Optimized immunotherapies to boost the potential anti-tumor T-cell response are still needed in CRC. A good candidate is the inflammasome pathway that bridges innate and adaptive immunity via the caspase-1/interleukin-18 (IL-18) axis, able to elicit a T-helper/cytotoxic (Th1/Tc1) anti-tumor response. This study aimed to determine the status of the caspase-1/IL-18 axis in tumor cells and its potential modulatory role on TILs in CRC. Using cohorts of CRC patients and an ex vivo explant culture model allowing functional and multiparametric approaches, we demonstrate that tumor cells in the majority of CRCs can be considered as innate immune cells that display a functional caspase-1/IL-18 axis able to modulate the adaptive Th1/Tc1 anti-tumor response of TILs. Furthermore, the identification of three distinct subgroups of CRC will provide a rationale for future strategies targeting the inflammasome pathway in CRC.

**Abstract:**

In colorectal cancer (CRC), a high density of T lymphocytes represents a strong prognostic marker in subtypes of CRC. Optimized immunotherapy strategies to boost this T-cell response are still needed. A good candidate is the inflammasome pathway, an emerging player in cancer immunology that bridges innate and adaptive immunity. Its effector protein caspase-1 matures IL-18 that can promote a T-helper/cytotoxic (Th1/Tc1) response. It is still unknown whether tumor cells from CRC possess a functional caspase-1/IL-18 axis that could modulate the Th1/Tc1 response. We used two independent cohorts of CRC patients to assess IL-18 and caspase-1 expression by tumor cells in relation to the density of TILs and the microsatellite status of CRC. Functional and multiparametric approaches at the protein and mRNA levels were performed on an ex vivo CRC explant culture model. We show that, in the majority of CRCs, tumor cells display an activated and functional caspase-1/IL-18 axis that contributes to drive a Th1/Tc1 response elicited by TILs expressing IL-18Rα. Furthermore, unsupervised clustering identified three clusters of CRCs according to the caspase-1/IL-18/TIL density/interferon gamma (IFNγ) axis and microsatellite status. Together, our results strongly suggest that targeting the caspase-1/IL-18 axis can improve the anti-tumor immune response in subgroups of CRC.

## 1. Introduction

The evolution of many solid tumors including colorectal cancer (CRC) is influenced by intricate and complex interactions between tumor and immune cells of the tumor microenvironment, depending on many different intrinsic factors including tissue-specific factors and genetic abnormalities of the tumor. These interactions lead to the development of a local immune response that can have immunosuppressive or immunostimulatory properties. Combining therapeutic strategies that can inhibit immunosuppression while activating tumor-specific T lymphocytes able to kill tumor cells currently represents the most promising therapeutic approach. In CRC, a high density of effector memory and cytotoxic tumor-infiltrating T lymphocytes (TILs) is positively correlated with a favorable prognosis [1,2,3,4]. In microsatellite instable (MSI) CRC, which accounts for 15% of all CRCs (as compared to microsatellite stable (MSS) CRC), recent evidence has shown the major role of a T-helper/cytotoxic (Th1/Tc1) gene signature in CRC immune surveillance, caused by frameshift and missense mutations leading to the formation of immunogenic neoepitopes and to an anti-tumor immune response [5,6]. In addition, we recently demonstrated that a preexisting in situ Th1/Tc1 immune response—identified by the expression of the transcription factor T-box expressed in T cells (Tbet)—positively impacted prognosis in MSI as well as in MSS CRC [7]. This anti-tumor immune response can be counterbalanced by inhibitory signaling pathways including immune checkpoint receptors (ICPs), and their ligands such as the programmed death receptor-1 (PD-1) or cytotoxic T lymphocyte-associated protein 4 (CTLA4) pathways [7,8], that can be blocked by specific antibodies defined as immune checkpoint inhibitors (ICIs). However, treatment with ICIs only leads to objective and durable responses in 30% to 50% of metastatic MSI CRC cases (mono-or combotherapy) and to a very low response rate in MSS CRC [9,10,11].

In this context, a better understanding of the immune contexture and of its modulation by tumor cells can lead to new therapeutic combinations in CRC, especially in MSS CRC. Approaches targeting some agents or signaling pathways bridging innate and adaptive immunity may expand the number of immunotherapy strategies able to restore or boost anti-tumor T cell responses. Among those, inflammasomes have recently emerged as players in cancer immunology and immunotherapy [12,13].

Inflammasomes, major components of innate immunity, are large multiprotein complexes that regulate the activation of the effector protein, caspase-1, expressed as an inactive precursor. Upon activation by pathogen- or damage-associated molecular patterns (PAMPs or DAMPs, respectively), active caspase-1 cleaves several substrates including gasdermin D (GSMD), pro-IL1β, and pro-IL-18 [14,15,16,17]. IL-18, originally termed IFNγ-inducing factor [18], is considered as a Th1-promoting cytokine since it elicits IFNγ production by T cells, which favors the generation and maintenance of a beneficial inflammatory microenvironment around tumor cells, with potential anti-tumor properties. However, IL-18 has been reported to have both anti-cancer and pro-cancer properties, depending on the organ studied and on the tumor environment. The mechanisms underlying these opposite effects are not yet clearly understood [12]. Besides being produced by immune cells including antigen presenting cells, IL-18 is expressed by epithelial cells and by cancer cells of some solid tumors such as melanoma, lung cancer, prostate, and colon cancer [19,20,21,22]. In lung cancer, IL-18 contributes to the expansion of CD8^+^ Tbet^+^ TILs that express IL-18 receptors and produce IFNγ within the tumor microenvironment [21]. Several sensors or regulators of inflammasomes, mainly studied in mouse models, have been reported to be expressed in cancer and to play distinct and sometimes opposite roles across tumor types, ranging from tumor promotion to tumor suppression [23]. Furthermore, the relative expression of inflammasome components may differ according to cell types, suggesting that they could exhibit distinct functions in different cell compartments depending on tissue-specific factors [23].

In the normal human colon, epithelial cells display both innate immune functions and immunomodulatory functions, essential for the maintenance of gut homeostasis. They constitutively express pro-IL-18 and pro-caspase-1 that can be rapidly activated upon stimulation by various bacterial or pro-inflammatory stimuli priming the inflammasome pathway and generating a mucosal Th1 (IFNγ) response, a driver of epithelial barrier disruption [24,25,26]. However, it is not known whether colonic cancer cells retain a functional caspase-1/IL18 pathway that could be activated and able to modulate the Th1/Tc1 response of TILs.

In this study, we used two independent cohorts of CRC patients to assess IL-18 expression and caspase-1 activation profiles in tumor cells and studied their relationship with TILs density and microsatellite status. In addition, functional and multiparametric approaches, at the protein and mRNA levels, were performed both on an ex vivo 3D model of CRC explant cultures that maintained cellular interactions able to build innate/adaptive immune responses (previously validated as a good preclinical model [7]), and on TILs isolated from CRC primary tumors.

## 2. Results

### 2.1. IL-18 Is Expressed by Tumor Cells in the Majority of CRCs and Correlates with the Density of Tbet^+^ or CD8^+^ Intraepithelial TILs

**Figure 1:**
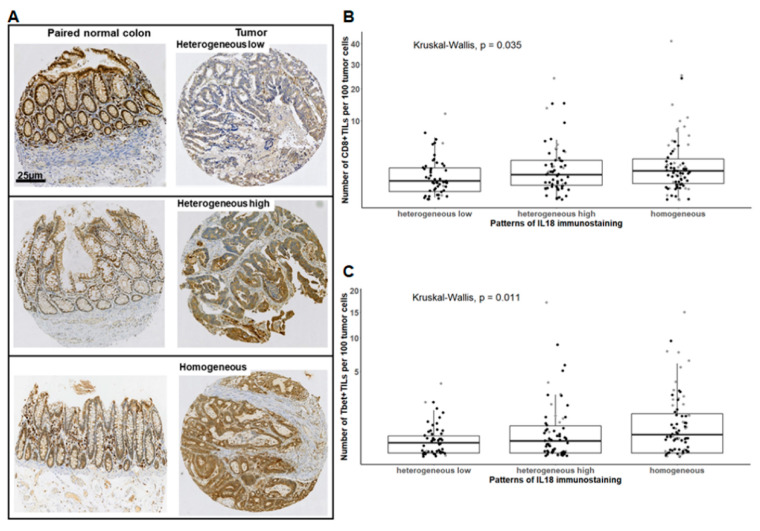
The interleukin-18 (IL-18) immunostaining pattern in tumor cells correlates with the density of CD8^+^ or Tbet^+^ intraepithelial tumor-infiltrating T lymphocytes (IEL-TILs) in colorectal cancer (CRC). (**A**) Representative examples of the IL-18 immunostaining pattern in tumor cells compared to paired normal epithelial cells, determined as mentioned in Materials and Methods. (**B**,**C**) Density of CD8^+^ or Tbet^+^ IEL-TILs according to IL-18 immunostaining pattern. Each dot represents the mean of triplicate counts for a given CRC. The square root function (sqrt) was used to transform Y axis values in order to visualize small value distributions. Black dots: Microsatellite stable (MSS) CRC; grey dots: microsatellite instable (MSI) CRC. Kruskal–Wallis test.

In the retrospective cohort of CRCs (*n* = 192), we first assessed the IL-18 immunostaining pattern using an antibody that detects both the proform and mature form of IL-18. In the normal colonic mucosa all epithelial cells highly expressed IL-18 throughout the colonic crypts (Figure 1A, left panel). The expression profile in tumor cells was compared to that of epithelial cells from paired normal colonic mucosa in terms of staining intensity and percentage of positive cells.

We identified three expression patterns of IL-18 in tumor cells. The “heterogeneous low” pattern included CRC with an IL-18 staining intensity lower in tumor cells than in the paired normal epithelial cells (10/192 = 5%) and CRC with an IL-18 staining intensity equal to that of paired normal epithelial cells in 10–49% of tumor cells (43/192 = 22%) (Figure 1A, upper panel). The other patterns were composed of CRCs with a staining intensity equal or higher than that of normal epithelial cells in 50–80% of tumor cells (“heterogeneous high”) (61/192 = 32) (Figure 1A, middle panel) or in 100% of tumor cells (“homogeneous”) (78/192 = 41%) (Figure 1A, lower panel). Thus, IL-18 was found to be highly expressed in tumor cells in 72% of CRCs. When considering the microsatellite status, 91% of MSI CRCs and 67% of MSS CRCs were found to express the highest percentage of IL-18-positive tumor cells. As IL-18 is known as a driver of the Th1/Tc1 response, we assessed intraepithelial Tbet^+^ and CD8^+^ TIL densities in relation to IL-18 expression in tumor cells. As shown in Figure 1B,C, CRCs with a “homogeneous” or “heterogeneous high” expression pattern of IL-18 were enriched in intraepithelial CD8^+^ and Tbet^+^ TILs compared to the “heterogeneous low” IL-18 expression pattern (*p* = 0.03 and 0.01, respectively).

Thus, these findings show that intracellular IL-18 detected by immunohistochemistry, i.e., mainly the proform, is maintained in tumor cells of most CRCs compared with the normal epithelial cells they derive from. In addition, IL-18 expression in tumor cells is associated with the Th1/Tc1 immune microenvironment, suggesting that IL-18 produced by tumor cells could influence the local immune contexture.

### 2.2. Active Caspase-1 Is Present in Tumor Cells in 70% of CRCs, and Positively Correlates with Up-Regulation of Inflammasome Sensors and Mature IL-18 Levels Secreted in Explant Cultures of CRC

**Figure 2:**
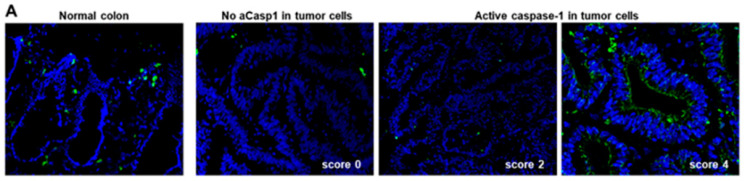

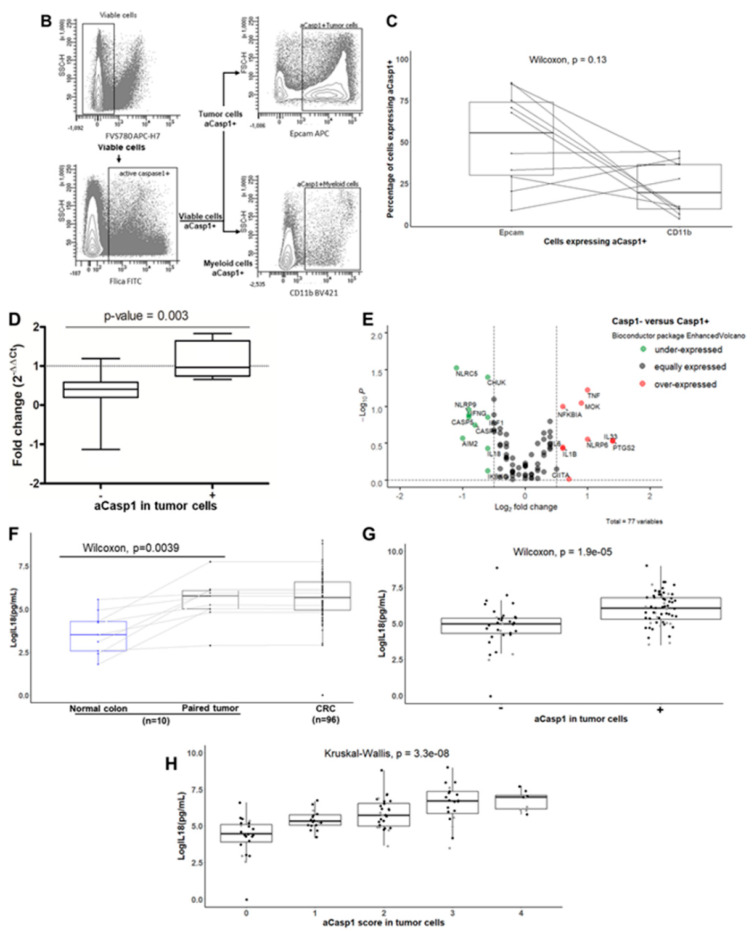
The presence of active caspase-1 (aCasp1) in tumor cells correlates with mature IL-18 levels secreted in explant cultures of CRC. (**A**) In situ detection of caspase-1 activity by the Figure 1. (membrane and cytoplasmic staining). Nuclei are stained with Dapi (blue). (**B**,**C**) Flow cytometry detection of caspase-1 activity on fresh CRC samples, ex vivo after mechanical dissociation, in Epcam+ tumor cells and CD11b+ myeloid cells. Gating strategy of a representative case (**B**); the histogram (**C**) shows the percentages of Epcam+ and CD11b+ cells expressing active caspase-1 (*n* = 10). (**D**) qPCR analysis of Caspase-1 mRNA levels in aCasp1^−^ (*n* = 8) versus aCasp1^+^ (*n* = 8) CRC relative to paired normal colon and normalized to S6. (**E**) Volcano plot of differentially expressed mRNA of inflammasome components using RT^2^ profiler qPCR array, in aCasp1^−^ (*n* = 8) versus aCasp1^+^ (*n* = 8) CRC. (**F**–**H**) Mature IL-18 secretion assessed by ELISA in the supernatants of 24-h explant cultures. Each dot represents the mean of triplicate cultures. Black dots: MSS CRC; grey dots: MSI CRC. Wilcoxon paired test (**F**) or unpaired test (**D**,**G**); Kruskal–Wallis test (**H**).

In order to assess whether pro-IL-18 expressed by tumor cells can be processed and secreted as a mature form, we first assessed the expression of active caspase-1, essential for the processing of IL-18. To this end, we used fresh tumor tissues from our prospective cohort of CRC patients (*n* = 96). Caspase-1 activity (“active caspase-1”) was detected in situ on unfixed frozen sections of the tumor in all cases of CRC (*n* = 96) and in the paired normal colonic mucosa at distance from the tumor as a control (*n* = 10), using the fluorescent inhibitor probe FAM-YVAD-FMK (FLICA (Fluorochrome Inhibitor of Caspases) assay) that binds to the active form of caspase-1. A semi-quantitative assessment of the percentage of tumor cells with active caspase-1 was performed (scores 0 to 4, see Materials and Methods section). As shown in Figure 2A, in the normal colonic mucosa, epithelial cells did not display active caspase-1; only a few subepithelial macrophages scored positive.

Considering the status of active caspase-1 (aCasp1) in tumor cells, two subgroups of CRC appeared. A minor group representing 30% (32/96) of CRCs, mainly MSS CRCs and only five MSI CRCs (i.e., 20% of MSI), displayed no or very low levels of active caspase-1 in tumor cells (<10% positive tumor cells, score 0–1) (Figure 2A). By contrast, active caspase-1 was detected in tumor cells in 70% (64/96) of CRCs, both MSS and the majority of MSI CRCs (*n* = 17, i.e., 80% of MSI CRCs). Active caspase-1 was located at the plasma membrane and/or in the cytoplasm (Figure 2A). In those aCasp1^+^ CRCs, the percentages of aCasp1^+^ tumor cells varied among CRCs from 10% to 70% aCasp1^+^ cells within tumor glands, with the following distribution—score 2 (10–30% positive cells): 35 cases; score 3 (30–50% positive cells): 20 cases; score 4 (50–70% positive cells): 9 cases.

In all CRCs, a few macrophages, located in the peritumoral areas and at the tumor invasive front, also expressed active caspase-1. To obtain a more quantitative assessment of aCasp1^+^ cells in the tumor microenvironment, we set up a multicolor flow cytometry panel combining a viability marker, caspase-1 FLICA assay, Epcam (for tumor cells), and CD11b (for myeloid cells) on cell suspensions obtained just after mechanical dissociation of fresh tumor fragments ex vivo (*n* = 10). The flow cytometry gating strategy is shown in Figure 2B, based on a representative case of CRC. Expression of active caspase-1 was observed predominantly in the Epcam^+^ tumor cells and at a much lower level in the CD11b^+^ myeloid cells in most of the cases studied (Figure 2B,C). Altogether, our results show that, contrary to the normal colonic mucosa, the majority of CRCs (70%) expressed active caspase-1, predominantly in tumor cells.

In order to assess whether the absence of active caspase-1 in some CRCs could be associated with changes in caspase-1 mRNA levels as previously reported [27], we assessed the relative expression of caspase-1 mRNA by qRT-PCR, in the tumor and paired normal colon in the two subgroups, aCasp1^+^ (*n* = 8) and aCasp1^−^ (*n* = 8). As shown in Figure 2D, the caspase-1 mRNA level was significantly down-regulated in aCasp1^−^ CRC compared with aCasp1^+^ CRC. To precisely obtain the whole inflammasome profile depending on the active caspase-1 status of CRC, we used the human inflammasome “RT^2^ profiler PCR array” to screen genes upstream and downstream of caspase-1 in the two CRC subgroups, aCasp1^−^ versus aCasp1^+^. Among the 84 genes screened, 7 genes were not detected, 58 were equally expressed in the two subgroups, 10 were under-expressed, and 9 were over-expressed in aCasp1^−^ versus aCasp1^+^ CRC. Consistently, under-expressed genes in aCasp1^−^ CRC subgroup included caspase-1, IL-18, and IFNγ (Figure 2E). Moreover, and interestingly, among inflammasome sensors, *AIM2*, *NLRP9*, and *NLRC5* were under-expressed in aCasp1^−^ CRC subgroup, whereas *NLRP6* was over-expressed (Figure 2E).

In order to verify whether the activation of caspase-1 in tumor cells was associated with secretion of mature IL-18, we assessed the link between the expression of aCasp1 in tumor cells and the levels of mature IL-18 released in culture supernatants of CRC explant cultures. Firstly, the level of IL-18 secreted in the tumor supernatants was compared with that measured in the supernatants of paired normal colonic mucosa (*n* = 10 cases). We found that the level of secreted IL-18 was significantly higher in the tumor than in paired normal colon (Figure 2F). When considering all cases of CRC (*n* = 96), we found that the level of IL-18 released in tumor explant cultures was heterogeneous among CRCs (Figure 2F) and correlated with the status of aCasp1 in tumor cells. Indeed, the level of IL-18 secreted was significantly higher in CRCs with aCasp1^+^ tumor cells than in those with aCasp1^−^ tumor cells (Figure 2G). In addition, a significant association was observed between IL-18 levels and the percentages of aCasp1^+^ tumor cells, irrespective of the microsatellite status of CRC (Figure 2H).

Altogether, our results demonstrate the presence of a “functional” inflammasome in tumor cells in the majority of CRCs (70%). However, unlike normal colonic epithelial cells they derive from, a variable proportion of tumor cells per tumor exhibits an aberrantly activated inflammasome (expression of active caspase-1 and release of mature IL-18), irrespective of the microsatellite status. Taking into account the association between IL-18 levels in tumor cells and the density of Th1/Tc1 TILs, our working hypothesis is that this “activated functional inflammasome” of tumor cells can facilitate the IFNγ response of TILs in CRC.

### 2.3. Relationship between Mature IL-18 and IFNγ Released in Ex Vivo Explant Cultures, and Effect of Recombinant Human IL-18 (rhIL18) on Isolated TILs That Express High Levels of IL-18Rα

**Figure 3:**
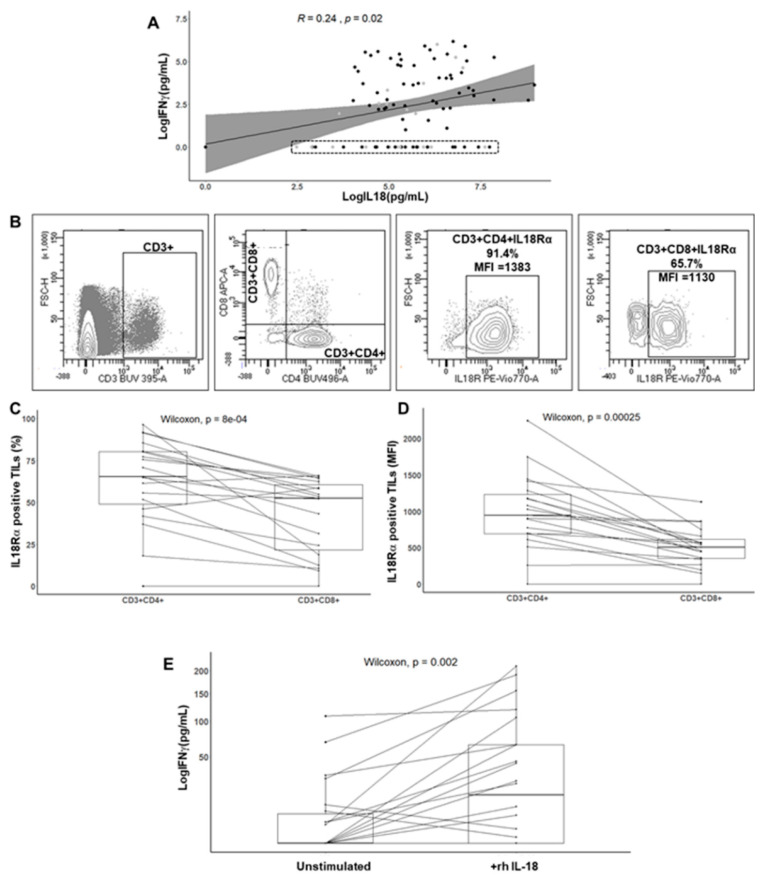
Relationship between IL-18 and interferon gamma (IFNγ) levels released in explant cultures and effect of recombinant human IL-18 (rhIL-18) on isolated TILs from CRCs expressing IL-18 receptor (IL-18Rα). (**A**) Correlation between the IFNγ response of TILs and mature IL-18 released in the CRC explant culture supernatants (*n* = 96). Spearman correlation test. Each dot represents the mean value of triplicate cultures. The dotted rectangle highlights IFNγ^−^ CRC (*n* = 33). (**B**) Expression of IL-18Rα by flow cytometry on TILs ex vivo. Gating strategy on a representative case. (**C**,**D**) Percentage and median fluorescent intensity (MFI) of IL-18Rα on CD3^+^CD4^+^ or CD3^+^CD8^+^ TILs isolated from CRC (*n* = 19). Wilcoxon paired test. (**E**) Effect of rhIL-18 (50 ng/mL, 24 h) on the IFNγ response of TILs cell lines from CRC (*n* = 20). Each dot represents the mean of triplicate cultures of TILs. The square root function (sqrt) was used to transform Y axis values in order to visualize small value distributions. Wilcoxon paired test.

To test this hypothesis, we first assessed the relation between mature IL-18 and IFNγ levels in the supernatants of CRC explant cultures (*n* = 96) by ELISA. As shown in Figure 3A, a significant association was found between IL-18 levels and the IFNγ response (*p* = 0.02). However, the IFNγ response was quite heterogeneous among patients, and two subgroups of CRC were isolated depending on the IFNγ levels. We found an expected subgroup with high IL-18 levels and moderate to high IFNγ levels (*n* = 63/96 (65%); 52 MSS (70%) and 11 MSI (50%)) and an unexpected subgroup featuring no IFNγ response (undetectable levels in triplicate cultures) despite moderate to high IL-18 levels (*n* = 33/96 (34%); 22 MSS (30%) and 11 MSI (50%)).

Then, we assessed the effect of recombinant human IL18 (rhIL-18) on the IFNγ response of TILs isolated from CRCs (*n* = 19), since IL-18 is known to stimulate IFNγ production [16]. We first measured the expression level of IL-18 receptors (IL-18Rα) on the CD3^+^ CD4^+^ and CD3^+^ CD8^+^ subsets ex vivo from cell suspensions obtained just after mechanical dissociation of fresh tumor fragments (*n* = 19; 11 MSS and 8 MSI CRCs). Flow cytometry gating strategy is shown in Figure 3B based on a representative case of CRC. In all cases except one, TILs expressed IL-18Rα in a variable and often high proportion and with a high MFI in most cases. In addition, the IL-18Rα level was higher in the CD4^+^ subset as compared to the CD8^+^ subset for a given CRC, both in terms of frequency and median fluorescence intensity (MFI) (Figure 3C,D). Finally, the frequency of IL-18Rα on TILs was not statistically different in MSS compared with MSI CRC. Finally, to test whether stimulation of IL-18 receptors could induce IFNγ production by TILs, we added rhIL-18 to the media of TILs expanded in culture from tumor fragments for 20 days (*n* = 20). While expanding TILs from tumor fragments, the IL-18Rα expression was maintained and stable. As shown in Figure 3E, a 24-h treatment of TILs with rhIL-18 (50 ng/mL) led to a significant increase in the baseline IFNγ level measured by ELISA (*p* = 0.002). In addition, the IFNγ fold increase was related to the IL-18Rα expression level assessed by flow cytometry.

Thus, our findings show that the majority of CRC exhibits an activated functional caspase-1/IL-18 axis in tumor cells, and that IL-18 is able to modulate the IFNγ response of TILs in vitro. However, one-third of CRCs, despite expressing an activated functional inflammasome and being infiltrated by Th1/Tc1 TILs, did not respond to IL-18 in terms of IFNγ secretion based on ex vivo explant cultures. These findings strongly suggest the existence of subgroups of CRC according to the entire caspase-1/IL18-IFNγ axis and microsatellite status.

### 2.4. Identification of Subgroups of CRC according to the Caspase-1/IL-18/TIL Density/IFNγ Axis and Microsatellite Status

We performed a non-hierarchical cluster analysis, in order to stratify the CRC patients according to the entire axis caspase-1/IL-18/TILs density/IFNγ axis, and to their microsatellite status. First, we used the Gower metric to assess the similarity/dissimilarity between CRCs according to the following mixed variables (both categorical and numerical): microsatellite status (MSS, MSI), aCasp1 status in tumor cells (present (+) or not (−)), levels of mature IL-18 and of IFNγ secreted in explant cultures, and density of TILs (CD8/CD3, Tbet/CD3, PD1/CD3 ratios).

**Figure 4:**
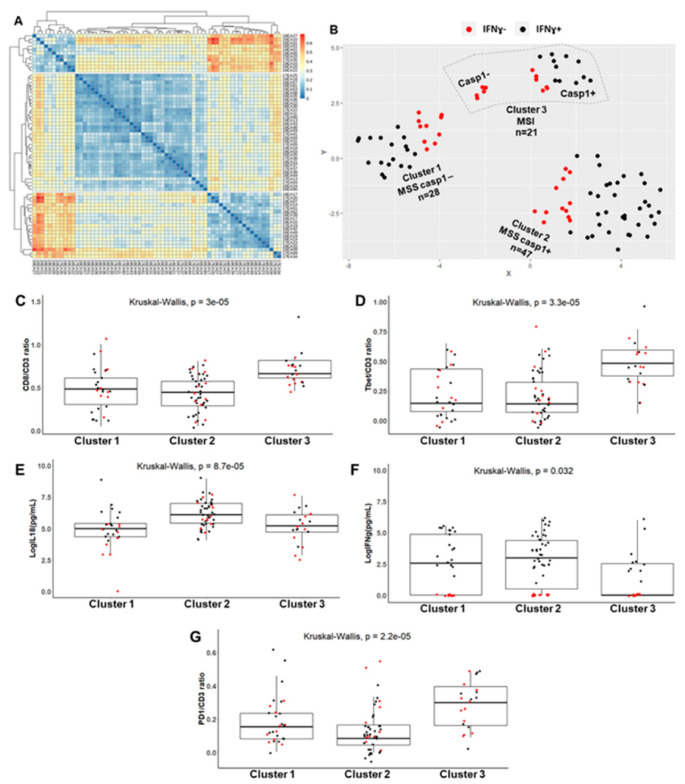
Identification of CRC clusters according to the caspase-1/IL-18/TIL density/IFNγ axis and microsatellite status. (**A**) Heatmap on the dissimilarity matrix based on the Gower metric. (**B**) Cluster visualization using a lower dimensional space with *t*-distributed stochastic neighborhood embedding (*t*-SNE). (**C**–**G**) Numerical variables distribution in the three CRC clusters: TIL density (**C**,**D**,**G**); IFNγ and IL-18 levels assessed by ELISA in CRC explant cultures (**E**,**F**). Each dot represents the mean value of triplicate explant cultures or TIL counts. Black dots: CRC with IFNγ response (IFNγ^+^); red dots: CRC without IFNγ response (IFNγ^−^).

Figure 4A shows the heatmap representing the similarity/dissimilarity matrix that allowed for the assessment of cluster tendency. Subsequently, a non-hierarchical cluster analysis was performed using partitioning around medoids (PAM), and silhouette analysis was used to choose the optimal number of clusters, i.e., three clusters. *t*-distributed stochastic neighbor embedding (*t*-SNE) two-dimensional projection was used to plot the different clusters. Figure 4B shows the three different clusters: cluster 1, featuring an MSS phenotype with aCasp1^−^ tumor cells (*n* = 28; 29%); cluster 2, featuring an MSS phenotype with aCasp1^+^ tumor cells (*n* = 47; 49%), and cluster 3, featuring mainly an MSI phenotype with aCasp1^+^ tumor cells (*n* = 16; 17%) and only five MSI cases with aCasp1^−^. The CRCs without IFNγ response (IFNγ^−^ red dots on Figure 4B) were more common in cluster 1 (36%, MSS aCasp1^−^) and cluster 3 (50%, MSI), than in cluster 2 (25%, MSS aCasp1^+^). Similar results were obtained using hierarchical clustering (Figure S1).

When examining the numerical variables among clusters, a significantly higher density in CD8^+^ or Tbet^+^ TILs was observed in cluster 3 (MSI) as compared with clusters 1 and 2 (MSS), as expected (Figure 4C,D). In addition, the levels of mature IL-18 were significantly higher in cluster 2 (MSS with active caspase-1) than in clusters 1 and 3 (MSS, no active caspase-1 and MSI) (Figure 4G). IFNγ levels were the lowest in cluster 3 despite the presence of active caspase-1 in tumor cells and high IL-18 levels (Figure 4F). One possible explanation for this finding would be the high density of PD1^+^ TILs in cluster 3 (Figure 4E).

The Consensus Molecular Subtypes (CMS) classification could be determined in 39 CRCs (11 CRCs in cluster 1, 20 in cluster 2, and 8 in cluster 3) using 3′-Seq Illumina. As shown in Figure S2 and as expected, CRCs of cluster 3 (MSI CRC) belonged to the CMS1 subgroup. CRCs of cluster 1 and 2 (MSS aCasp1^−^ or aCasp1^+^, respectively) both belonged mostly to the CMS2 subgroup.

Altogether, this study (1) identifies several clusters of CRCs according to the caspase-1/IL-18/TIL density/IFNγ axis, and microsatellite status, and (2) points out a “paradoxical” subgroup (34% of CRCs) present in all clusters, mostly in clusters 1 and 3, that does not display an IFNγ response despite the moderate to high levels of mature IL-18.

### 2.5. Distinctive Features of the IFNγ^+^ and IFNγ^+^ Subgroups

**Figure 5:**
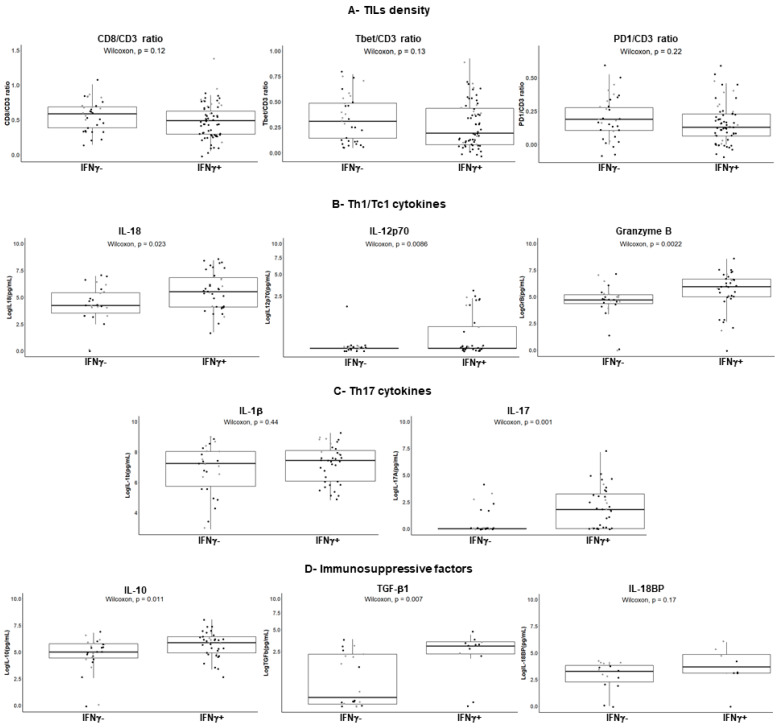
Features of the IFNγ^−^ and IFNγ^+^ subgroups of CRC. (**A**), Density of TILs (*n* = 96) according to their IFNγ response. Results are expressed as the CD8/CD3 ratio, Tbet/CD3 ratio, or PD1/CD3 ratio after immunohistochemistry on serial sections and counts of total TILs (intra- and peri-tumoral) using Imaris software. Each dot represents the mean value of triplicate counts. Wilcoxon unpaired test. (**B**–**D**) (left), Cytokine profiles assessed by a bead-based multiplex immunoassay in explant culture supernatants. IFNγ^+^ (*n* = 36); IFNγ^−^ (*n* = 26). Transforming Growth Factor beta 1 (TGFβ1) (*n* = 32) and IL-18 binding protein (IL-18BP) (*n* = 23) levels were assessed by ELISA (**D**, middle and right). Each dot represents the mean of duplicate cultures for a given CRC. The square root function (sqrt) was used to transform Y axis values in order to visualize small value distributions (IL-12p70, TGFβ1). Wilcoxon unpaired test. Black dots: MSS CRC; grey dots: MSI CRC.

We further investigated the potential distinct features between the IFNγ^−^ versus IFNγ^+^ subgroups of CRC in terms of TIL density and secreted cytokines, as well as immunomodulatory factors such as PD1, IL-10, and Transforming Growth Factor beta 1 (TGFβ1), known to suppress the IFNγ response. We first examined the TIL density in the IFNγ^+^ and IFNγ^−^ subgroups of CRC. As shown in Figure 5A (upper panel), there was no significant difference in terms of either the density of CD8^+^ or Tbet^+^ TILs or of TILs expressing the immune checkpoint PD1.

Then, we performed a multiplex bead-based assay (13-plex) for simultaneous detection of Th1/Tc1 cytokines and mediators, as well as Th17 and anti-inflammatory cytokines in the culture supernatants of 62 CRCs for which sufficient amounts of remaining supernatant were available prior the assay (*n* = 26 IFNγ^−^ CRC and *n* = 36 IFNγ^+^ CRC). As shown in Figure 5B and as expected, the Th1 cytokine and Tc1 marker granzyme B levels were higher in the IFNγ^+^ compared to the IFNγ^−^ subgroups. With respect to the IFNγ-inducing cytokines IL-18 and IL-12p70, levels were higher in the IFNγ^+^ subgroup; IL-12p70 was detectable in only 30% of IFNγ^+^ CRCs. Interestingly, IL-17A, a prototypic Th17 cytokine, was undetectable in most of the IFNγ^−^ subgroup (Figure 5C), suggesting that TILs, although present, were not functional in this subgroup. Therefore, we assessed the anti-inflammatory cytokines (IL-10, TGFβ1) that could suppress the IFNγ response and found that they were not secreted at higher levels in the IFNγ^−^ subgroup (Figure 5D). Finally, we assessed by ELISA the secretion of IL-18 binding protein (IL-18BP), a natural inhibitor of IL-18, part of a negative feedback loop, that can act as an immunosuppressive molecule [28,29]. As shown in Figure 5D (right), IL-18BP was not overexpressed in the IFNγ^−^ subgroup.

**Figure 6:**
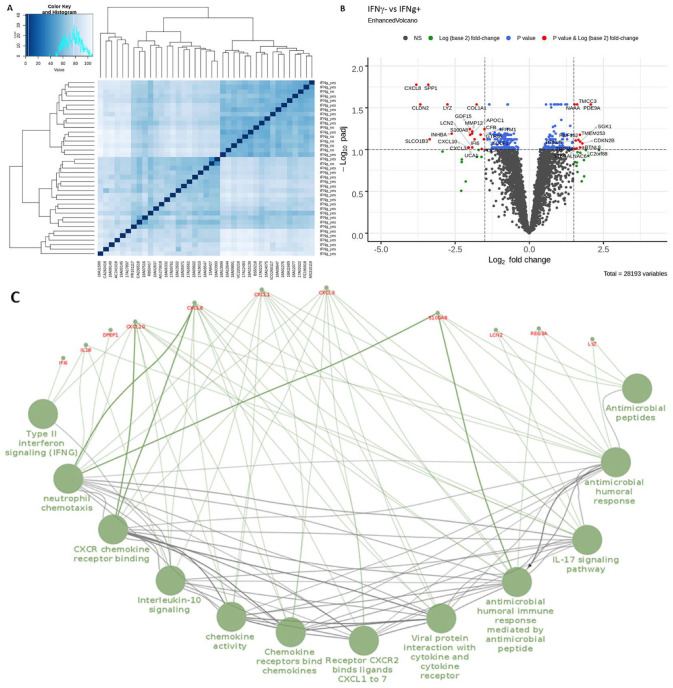
Differentially expressed genes (DEGs) between the IFNγ^−^ versus IFNγ^+^ subgroups of CRC (IFNγ^+^ (*n* = 32); IFNγ^−^ (*n* = 7)). (**A**) Heatmap of Euclidean sample distance after rlog transformation using the DESeq2 package. (**B**) Volcano plot of DEGs using the “EnhancedVolcano” package. (**C**) Functionally organized network of enriched pathway using ClueGO and Cluepedia plugins.

To further explore the features of the IFNγ^−^ subgroup, we performed a transcriptomic analysis of the IFNγ^+^ and IFNγ^−^ CRC using 3′-Seq Illumina of 39 CRC (32 IFNγ^+^ and 7 IFNγ^−^). Heatmap of Euclidean sample distances after Log transformation shows a homogeneous IFNγ^−^ subgroup, while the IFNγ^+^ subgroup was heterogeneous (Figure 6A). Figure 6B illustrates the most significant differentially expressed genes after *p*-value adjustment using the Benjamini and Hochberg method with at least an absolute log2 fold change of 1.5 (11 upregulated and 21 downregulated genes, based on the adjusted *p* value), in the IFNγ^−^ versus IFNγ^+^ subgroup (considering IFNγ^+^ as control group). The IFNγ^−^ subgroup was mainly characterized by downregulated genes such as chemokines (*CXCL3*, *CXCL8*, *CXCL10*) and antimicrobial agents (*S100A8*, *REG3A*, *LYZ*, *LCN2*) (Figure 6B). We next performed pathway enrichment analysis of downregulated genes in the IFNγ^−^ subgroup since the upregulated genes were not associated with any signaling pathway. To this end, we used ClueGO, a Cytoscape plug-in which is continuously updated, that integrates many biological information from different databases such as Gene Ontology (GO), Kyoto Encyclopedia of Genes and Genomes (KEGG), Reactom, and BioCarta. Those downregulated genes were significantly associated with specific signaling pathways related to innate and adaptive immunity such as anti-microbial peptides, neutrophil chemotaxis and migration, chemokines, IFNγ signaling, and IL-17 signaling (Table S1). In order to highlight these enriched pathways, we used ClueGO to visualize an organized network and the functional links between those downregulated genes (Figure 6C). Logically, the IFNγ signaling pathway was downregulated in the IFNγ^−^ subgroup, together with the IL-17 and IL-10 signaling pathways, a finding in accordance with our results at the protein level.

## 3. Discussion

The main findings of this study, based on a multiparametric approach (both descriptive and functional) using freshly dissociated primary tumors and 3D explant cultures of CRC patients, are multiple. Firstly, in the majority of CRCs (70%), tumor cells maintained a functional but aberrantly activated caspase-1/IL-18 axis compared with paired normal colonic epithelial cells. Secondly, this caspase-1/IL-18 axis was able to modulate the IFNγ response of resident Th1/Tc1 TILs. Thirdly, three clusters of CRC were identified according to the status of their caspase-1/IL-18/TIL density/IFNγ axis and microsatellite status. Finally, the existence of a “paradoxical” subgroup of CRC (34% of CRCs) was identified that does not display an IFNγ response despite the secretion of mature IL-18 by tumor cells and that features downregulated IL-17, chemokines, and antimicrobial agent gene signatures.

First, in the retrospective cohort of CRC patients, we showed herein that intracellular IL-18 detected by immunohistochemistry, i.e., mainly the proform, was maintained in tumor cells in the majority of CRCs compared to the paired normal colonic epithelial cells they derived from, although at variable levels in terms of number of positive cells within the tumor and staining intensity. Interestingly, in 90% of MSI CRCs and 70% of MSS CRCs, an intense IL-18 immunostaining was observed in more than 50% of tumor cells, which was associated with a higher density of intraepithelial CD8^+^ and Tbet^+^ TIL (IEL-TIL). These findings suggest that IL-18 produced by tumor cells can influence the density of the IEL-TIL infiltrate that we and others previously reported to be higher in MSI and a subgroup of MSS CRCs. In addition, these IEL-TILs positively impact overall survival and progression-free survival in our cohort (not shown), consistently with previous studies in CRC [2,7]. In our study, IL-18 was lost in tumor cells in 5% of CRCs and heterogeneously expressed in 10–40% of tumor cells in about 20% of CRC that did not show any distinct clinicopathological or molecular features. This partial or complete loss of IL-18 protein expression in tumor cells could be accounted for by a decrease or loss of IL-18 transcripts, as suggested by our transcriptional analyses on some CRCs of the prospective cohort and as previously reported [19,22,27]. This loss of mRNA expression levels could result from some polymorphisms in the IL-18 gene, previously described in colon cancer [30] and that have been associated with gastrointestinal cancer risk [31].

One can ask whether IL-18 can be considered as a prognostic factor in CRC, as suggested in a small number of CRC patients [19]. In our study, the expression pattern of pro-IL-18 expression by tumor cells did not significantly influence overall survival in univariate or multivariate analyses (not shown), a finding consistent with a previous study performed in silico using cancer gene expression databases [22]. These apparently contradictory results highlight the need to look for the mature biologically active form of IL-18.

The next step was to assess whether tumor cells possessed the enzymatic machinery essential for the cleavage of pro-IL-18 into its mature form, i.e., active caspase-1, which is tightly regulated by the inflammasomes (NLRP or NLRC). We designed an experimental approach on fresh tissues allowing us to assess caspase-1 activity, in situ and by flow cytometry using the FLICA assay, in relation with inflammasome-dependent cytokines (IL-18, IFNγ) secreted in the supernatants of ex vivo explant cultures of the tumor and with TIL density/phenotype. To our knowledge, our findings are the first to show the presence of two subgroups of CRC: a minor subgroup devoid of active caspase-1 (aCasp1^−^) in tumor cells (an expression profile close to that of normal colonic epithelial cells), and a major subgroup with aberrantly active caspase-1 in tumor cells (aCasp1^+^). A minor subgroup of CRCs (30%) (aCasp1^−^ in tumor cells), mainly MSS CRCs, secrete “basal” mature IL-18 levels close to those of normal colon explant cultures, released by a few activated aCasp1^+^ macrophages. Interestingly, the absence of caspase-1 activity in tumor cells was not only associated with decreased mRNA levels of caspase-1 and downstream cytokines IL-18 and IFNγ, but also with upstream inflammasome sensors or regulators, among which NLRC5 is of particular interest. Indeed, NLRC5 has been involved in the formation of a functional inflammasome [32] and more recently in the regulation of adaptive immune responses in several cell types via its role as an MHC class I transactivator [33,34]. Its status in human colon cancer and its role in the modulation of the immune response in CRC are as yet unknown; studies are underway to explore this issue.

In the major subgroup of CRCs (70% of CRCs, 63% of the MSS and 80% of the MSI CRCs), tumor cells exhibited an aberrantly active caspase-1 (compared to paired normal colonic epithelial cells), which was functional since the percentage of aCasp1^+^ tumor cells positively correlated with the amount of mature IL-18 secreted in the supernatants of CRC explant cultures. Noticeably, flow cytometry experiments confirmed that active caspase-1 was present predominantly in Epcam^+^ tumor cells and in only a few CD11b^+^ myeloid cells.

Together, our findings showed that the presence of an activated caspase-1/IL-18 axis in tumor cells in the majority of CRCs led to the secretion of mature IL-18. At this stage, our working hypothesis was that IL-18, which influences the density of Tbet^+^ TILs (our study and [22]), can facilitate the IFNγ response elicited by resident Th1/Tc1 TILs. This hypothesis was supported by our demonstration that (1) TILs isolated from CRC, mostly CD4^+^ and half of the CD8^+^ subset, expressed IL-18 receptors, and (2) IL-18 stimulation significantly improved the basal IFNγ production of TILs, an increase correlated with the IL-18R expression level and occurring without T cell receptor (TCR) activation. This hypothesis was also sustained by our findings using ex vivo explant cultures whereby TCR activation could occur via retained cellular interactions in this model, showing a significant association between IL-18 and IFNγ levels in 65% of CRCs.

Our clustering analysis identified three clusters of CRCs according to the status of their caspase-1/IL-18/TIL density/IFNγ axis and microsatellite status. As expected, MSI CRCs were grouped in a single cluster (cluster 3), and nearly all the cases found in this cluster belonged to the CMS1 subtype of the Consensus Molecular Subtypes (CMS) classification, featuring a “MSI, immune phenotype” [35]. We demonstrate for the first time in this study that this cluster is mostly characterized by aCasp1^+^ tumor cells and production of mature IL-18 associated with the highest level of Tbet^+^ and PD1^+^ TILs, possibly resulting in TIL exhaustion, in accordance with the lowest level of IFNγ. Furthermore, the clustering analysis identified two distinct clusters of MSS CRCs (cluster 1 (38% of MSS CRCs) and cluster 2 (62% of MSS CRCs)) that both belong mostly to the CMS2 subtype, without any distinct clinicopathological features. Contrary to the MSI CRC of cluster 3, MSS CRC of clusters 1 and 2 displayed an IFNγ response in relation with the presence of Tbet^+^ PD1^−^ TILs. However, the major differences between cluster 1 and cluster 2 was the tumor cell inflammasome status (aCasp1^−^ tumor cells in cluster 1 and aCasp1^+^ tumor cells in cluster 2) and the IL-18 level, higher in cluster 2 than in cluster 1. These findings suggest that the baseline IFNγ level found in cluster 1 is independent of the caspase-1/IL18 axis and that the activation of the caspase-1/IL18 axis in tumor cells could facilitate the anti-tumor IFNγ response. Further studies are needed to explore the stimuli able to activate caspase-1 in tumor cells of CRC. In addition, the 5-year follow-up of patients in our prospective cohort that will soon take place will determine whether a functional caspase-1/IL-18 axis can be a favorable prognostic marker in CRC.

Finally, and unexpectedly, we identified a “paradoxical” subgroup of CRC (*n* = 33/96 (34%)) that did not display any IFNγ response although they produced mature IL-18 and featured aCasp1+ tumor cells in 50% of cases. We showed that this absence of IFNγ response in CRC, regardless of the microsatellite status, could not be accounted for by (1) a significant decrease in CD8^+^, Tbet^+^ TIL infiltrate, (2) the absence of IL-18Rα expression by those TILs, (3) the overexpression of the immune checkpoint PD1 as well as of other ICPs (personal observation), or (4) an increase in the main immunomodulatory cytokines (IL-10, TGFβ) in the tumor microenvironment. Another explanation for the absence of IFNγ response in this paradoxical subgroup could be the increased expression of the natural inhibitor of IL-18, the so-called IL-18 binding protein (IL-18BP), that represents a potential mechanism of tumor escape from Th1 responses and is now considered as a new immune checkpoint [28,29]. However, our preliminary results indicate that IL-18BP is not over-secreted in the IFNγ^−^ versus IFNγ^+^ subgroup.

This absence of IFNγ secretion could also be due (downstream from IL-18R) to epigenetic or post-transcriptional alterations of IFNγ such as hypermethylation, as previously reported in solid tumors including CRC [36,37]. Interestingly, in our study, the lack of IFNγ response was strongly associated with decreased IL-17A secretion in accordance with down-regulation of the IL-17 signaling pathway at the transcriptomic level. IL-17 is well known to orchestrate the immune response at sites most exposed to microorganisms [38], such as the colonic mucosa. Besides, this down-regulation of the IL-17 signaling pathway was associated with a significant down-regulation of many genes such as those of chemokines, chemokine receptors, neutrophil chemotaxis and migration, and bacterial/viral immune responses. These are known to be involved in immune surveillance, bridging innate and adaptive immune responses associated with a “hot” tumor phenotype and modulating the inflammasome pathway [39,40]. These findings highlight the crucial role of innate immunity (at least with regard to the tumor cell inflammasome that can be triggered by gut microbiota) in facilitating an effective adaptive anti-tumor immune response. Further studies are needed to more precisely explore the complex interactions between tumor cells and the microbiota in CRC.

## 4. Materials and Methods

### 4.1. Patients

This study included 288 CRC patients from two independent cohorts, a retrospective cohort (*n* = 192) and a prospective one (*n* = 96). All tissues were processed according to the Helsinki Declaration and the guidelines of the French ethics committee for research on human tissues. The institutional board of Nantes University Hospital approved the study. Tissue biocollection was registered with the French Ministry for Higher Education and Research (DC-2014-2206) with approval from the ethic committee (CPP Ouest IV-Nantes). Each patient included in this study signed an informed consent form. Table 1 summarizes the clinicopathological features and microsatellite status of CRC patients, as well as the study design with respect to the two cohorts.

### 4.2. Tissue Microarray (TMA) Construction

For the retrospective cohort, TMA construction was performed as previously described [41]. Briefly, three representative areas of tumor and one area of paired normal colonic mucosa per patient were selected from hematoxylin–eosin-stained sections, and the tissue cores (1 mm in diameter) were inserted in a recipient paraffin block.

### 4.3. Quantitative Immunohistochemistry (IHC)

Here, 3-µm paraffin sections of TMA blocks from the retrospective cohort and of a representative tumor block from the prospective cohort were labeled with the following primary antibodies: IL-18 (10 µg/mL, polyclonal rabbit antibody, MBL), CD8 (3 µg/mL, clone C8/144B, Agilent), Tbet (2 µg/mL, clone 4B10, Santa Cruz Biotechnology, Heidelberg, Germany), CD3 (6 µg/mL, polyclonal rabbit antibody, Agilent, Santa Clara, CA, USA), and PD1 (2.5 µg/mL, clone NAT105, Abcam, Cambridge, UK) on an automated stainer (AutostainerLink 48, Agilent) according to a standard protocol. The immunological reaction was visualized using Peroxidase/DAB Envision system (Agilent) and sections were counterstained with hematoxylin. After IHC, slides were scanned using a NanoZoomer (Hamamatsu Photonics, Massy, France).

The number of CD8^+^ and Tbet^+^ intraepithelial-TILs (IEL-TILs) were counted per 100 tumor cells on TMA from the retrospective cohort using the ImageJ software, on the three areas of tumor per patient. Concerning the prospective cohort, we assessed the number of CD3^+^, CD8^+^, Tbet^+^, and PD1^+^ intraepithelial TILs as well as TILs of the stroma (per 100 tumor cells or stromal cells) using Imaris software. Cell counts were performed on three fields of a section of a given tumor. Results were expressed as the mean of the three counts.

Scoring of IL-18 expression patterns in tumor cells, performed on TMAs of the retrospective cohort, was done on the basis of the staining intensity of tumor cells compared with that of paired normal colonic epithelial cells and of the percentage of positive tumor cells. Three expression patterns were identified: a “heterogeneous low” pattern with a staining intensity lower in tumor cells than in paired normal epithelial cells or equal to that of normal epithelial cells in 10–49% of tumor cells; a “heterogeneous high” pattern with a staining intensity equal or higher than in normal epithelial cells in 50–80% of tumor cells; and a “homogeneous” pattern with a staining intensity equal or higher than in normal epithelial cells in 100% of tumor cells. The TMA sections were scored by two independent observers.

The microsatellite status of CRC was assessed by IHC using anti-MLH1 (clone E505), MSH2 (clone FE11), MSH6 (clone EP49), and PMS2 (clone EP51) antibodies, as previously described [7].

### 4.4. Active Caspase-1 Detection in Tumor Cells

Active caspase-1 was detected on unfixed frozen (8 µm) sections of tumor fragments of the prospective cohort (*n* = 96) and of some paired normal colonic mucosa (*n* = 10), using the fluorescent inhibitor probe FAM-YVAD-FMK (1:150, 1h at room temperature) that binds specifically to the active form of caspase-1, using the FAM FLICA Caspase-1 Assay Kit (ImmunoChemistry Technologies, Bloomington, IN, USA). Nuclei were counterstained with Dapi (1:1000); sections were post-fixed with 10% paraformaldehyde, washed twice, and mounted in ProLong Gold Antifade medium (Invitrogen, Carlsbad, CA, USA). Slides were imaged using a confocal microscope (Nikon A1-SIM). A semi-quantitative assessment of the percentage of active caspase-1 positive tumor cells (aCasp1^+^) was performed by two independent observers, on three consecutive fields inside the tumor. The scores were as follows: score 0 (absence of aCasp1 in tumor cells); score 1 (≤10% aCasp1^+^ in tumor cells); score 2 (10–30%); score 3 (30–50%) and score 4 (>50% aCasp1^+^ in tumor cells).

### 4.5. Ex Vivo CRC Explant Cultures of Prospective Cohort

Three fresh fragments of tumor (30 mg each) per CRC patient of the prospective cohort (*n* = 96) were maintained in culture for 24 h, as previously described [7]. In parallel, in some cases (*n* = 10), three paired normal tissue samples (30 mg) at a distance from the tumor were also cultured for 24 h. The supernatants of all explant cultures were collected, centrifuged, and stored at −80 °C for further assessment of cytokines (see below).

### 4.6. Cytokine Assays in the Supernatants of CRC Explant Cultures

The following cytokines were measured by ELISA in the supernatants of 24-h CRC explant cultures: IFNγ (Diaclone, Besançon, France), IL-18 (Bio-Techne, Minneapolis, MN, USA), IL-18BP (Bio-Techne), and TGFβ1 (Biolegend, San Diego, CA, USA), according to the manufacturer’s instructions. In addition, in 62 CRCs, a simultaneous detection of pro- or anti-inflammatory cytokines and cytotoxic factors was performed using a bead-based multiplex immunoassay technique (Custom 13-plex LEGENDplex^TM^ multianalyte flow assay kit, Biolegend). The following cytokines and factors were assessed: IFNγ, IL-18, IL-12p70, granzyme B, IL-1β, IL-17A, and IL-10. Data were acquired on a Canto II HTS flow cytometer (BD Biosciences, San Jose, CA, USA) and analyzed using the Legendplex data analysis software. Results were first expressed as pg/mL and then normalized using the natural log (ln) transformation.

### 4.7. Generation of TILs Cell Lines, Culture and Treatment with rhIL-18

TILs from 22 CRC patients were obtained after culture of tumor fragments for three weeks in RPMI 1640 medium supplemented with 8% human serum, antibiotics, and 150 U/mL IL-2, as previously described [41]. A weekly check for mycoplasma absence was performed using the HEK-Blue Detection Kit (InvivoGen, San Diego, CA, USA). Then, the obtained TIL cell lines were cultured for 24 h in the presence or absence of recombinant human IL-18 (rhIL-18; B001-5 (MBL, Woburn, MA, USA); 50 ng/mL; 1 × 10^6^ cells in 12-well plates) and IFNγ secreted in culture supernatants was assessed by ELISA as mentioned above.

### 4.8. Flow Cytometry Analysis

Fresh tumor tissue was minced with scissors and mechanically dissociated in a non-enzymatic solution using the GentleMACS dissociator (Miltenyi Biotec, Bergisch Gladbach, Germany) as previously described [41]. To precisely identify the cell types expressing active caspase-1, 5 × 10^5^ cells were incubated for 30 min at 37 °C with the fluorescent probe FAM-YVAD-FMK (1:150) in Dulbecco’s modified Eagle Medium (DMEM)/10% fetal Bovine serum (FBS), and washed twice with Phosphate Buffered Saline (PBS)/0.1% Bovine Serum Albumin (BSA). Then the cells were incubated for 30 min at 4 °C in the presence of the viability dye FVS eFluor^TM^ 780 (BD Biosciences), and the following antibodies: Epcam-APC (Biolegend), anti-CD11b-BV421 (BD Biosciences), or the isotype control antibodies. To detect IL-18 receptors on TILs, 1 × 10^6^ cells were incubated for 30 min at 4 °C with FVS 780 (BD Biosciences) and the following antibodies: IL-18α PE-Vio 770 (Miltenyi Biotec), CD3-BUV395 (BD Biosciences), CD4-BUV496 (BD Biosciences), CD8-APC (Biolegend), or the isotype control antibodies. After washing twice with PBS/0.1% BSA, positive cells were acquired in the viable cell gate on a LSR Fortessa × 20 flow cytometer (BD Biosciences) and analyzed using BD FACS Diva software 8.0.2 version.

### 4.9. Transcriptomic Analysis

#### 4.9.1. RNA Extraction

Fresh samples of tumor and paired normal colonic mucosa were submerged in RNAlater (RNA stabilization reagent, Qiagen, Hilden, Germany) for 24 h at 4 °C and stored at −80 °C after RNAlater removal. Total RNA was extracted using the RNeasy mini kit (Qiagen) and DNase treatment, according to the manufacturer’s procedure, after tissue homogenization with Fastprep-24 (MP Biomedicals, Irvine, CA, USA). RNAs were quantified using a NanodropND-1000 spectrophotometer (Thermo Fisher Scientific, Waltham, MA, USA) and their quality was assessed using Agilent RNA 6000 Nano kit with a 2100 Bioanalyzer instrument according to the manufacturer’s protocol.

#### 4.9.2. Quantitative RT-PCR

cDNA was synthetized using 2 µg of total RNA with Maxima H Minus reverse transcriptase (Thermo Fisher Scientific). qPCR analysis of caspase-1 (forward primer 5′-GCCCACCACTGAAAGAGTGA-3′; reverse primer 5′-TTCACTTCCTGCCCAC AGAC-3′) and housekeeping gene S6 (forward primer 5′-GCCCCAAAAGAGCTAGCAGA-3′; reverse primer 5′-TAGCCTCCTTCATTCTCTTGGC-3′) was performed using Maxima SYBR Green/ROX qPCR Master mix (Thermo Fisher Scientific) with a Mx3005P real-time PCR System (Agilent Technologies, Santa Clara, CA, USA). Thermal cycling was: one step at 95 °C for 10 min, followed by 40 cycles at 95 °C for 15 s and 60 °C for 1 min. Melting curve analysis was performed with a temperature gradient of 70–95 °C to circumvent potential nonspecific amplification. Relative caspase-1 expression in the tumor and paired normal colon was calculated from duplicate values using the ΔΔCt method. Data were first normalized to the housekeeping gene S6 and then caspase-1 mRNA levels in the tumor were expressed relative to those of paired normal colon. The human inflammasome RT^2^ profiler PCR array (Qiagen) was used to identify genes of the inflammasome pathway. To this end, 2 μg of total RNA from tumor samples of 16 CRC were reverse-transcribed using the RT^2^ First Strand Kit (Qiagen) and qPCR was performed using the RT^2^ SYBR Green ROX qPCR Mastermix (Qiagen) according to the manufacturer’s instructions. PCR array data were analyzed using The GeneGlobe Data Analysis Center web portal from Qiagen.

#### 4.9.3. 3′RNA Sequencing

The 3′RNA-seq was performed by the Genomics and Bioinformatics Core Facility (GenoBiRD, SFR Bonamy, Nantes, France) as previously reported [42]. The libraries were prepared from 10 ng of total RNA. The mRNA poly(A) tails were tagged with universal adapters, well-specific barcodes and unique molecular identifiers (UMIs) during template-switching reverse transcriptase. Barcoded cDNAs from multiple samples were then pooled, amplified and tagmented using a transposon-fragmentation approach (Nextera DNA Sample Prep kit, FC-121-1030, Illumina, San Diego, CA, USA) which enriches for 3′ends of cDNA. Size library was controlled on 2200 Tape Station Sytem (Agilent Technologies). A library of 350–800 bp length was run on an Illumina HiSeq 2500 using a Hiseq Rapid SBS Kit v2–50 cycles (ref FC-402-4022) and a Hiseq Rapid PE Cluster Kit v2 (ref PE-402-4002) according to the manufacturer’s protocol (Denaturing and Diluting Libraries for the HiSeq^®^ and GAIIx, Part # 15050107 v03 protocol, Illumina). Raw fastq pairs matched the following criteria: the 16 bases of the first read correspond to 6 bases for a designed sample-specific barcode and 10 bases for a unique molecular identifier (UMI). The second read (58 bases) corresponds to the captured poly(A) RNAs sequence. We performed demultiplexing of these fastq pairs in order to generate one single-end fastq for each of the 39 samples. These fastq files were then aligned with Burrow-Wheeler Aligner (BWA) to the reference mRNA refseq sequences and the mitochondrial genomic sequence, both available from the UCSC download site. Gene expression profiles were generated by parsing the alignment files (.bam) and counting for each sample the number of UMIs associated with each gene. Reads aligned on multiple genes, containing more than three mismatches with the reference sequence, or having a polyA pattern were discarded. Finally, a matrix containing the counts of all genes on all samples was produced. The expression values, corresponding to the absolute abundance of mRNAs in all samples, was then ready for further gene expression analysis. The analysis pipeline is available at https://bio.tools/3SRP. The R package DESeq2 [43] was used for differential analysis. Further analysis of pathway gene set enrichment was carried out using ClueGo, a Cytoscape plug-in that integrates Gene Ontology (GO) terms as well as KEGG/BioCarta pathways [44].

### 4.10. Statistical Analyses

Triplicates of TIL counts (CD3, CD8, Tbet, PD1) and cytokine levels (IL-18, IFNγ) were expressed as mean values after assessment of reproducibility using the estimation of the intraclass correlation coefficient. TIL counts were normalized using ratios to CD3; cytokine levels were normalized using the natural log (ln) transformation. Then, the relation between different parameters (numerical and categorical variables) was assessed using the Wilcoxon, Kruskal–Wallis, and Spearman non-parametric tests as appropriate. Multivariate analysis was used to analyze both categorical and numerical variables. Principal component analysis adapted for mixed data (both numerical and categorical variables, Factor Analysis of Mixed Data (FAMD)) was used to summarize and visualize the variance in the prospective cohort of CRC. Then, unsupervised clustering was used to highlight the existence of CRC subgroups by: (1) calculating distances between patients with the Gower metric; (2) partitioning around medoids (PAM), a classification method similar to k-means, but more robust to noise and outliers; and (3) selecting the right number of clusters with the silhouette width metric. Clusters were visualized using the *t*-distributed stochastic neighborhood embedding method (*t*-SNE). Plots were created using the ggplot2 package. Statistical analyses were performed using R software 3.6.0 and Rstudio software (version 1.2.1335). A *p* value less than 0.05 was considered as statistically significant.

## 5. Conclusions

In conclusion, this study showed that tumor cells of the majority of CRCs display a functional caspase-1/IL-18 axis, part of the inflammasome pathway, that can modulate the IFNγ response elicited by Th1/Tc1 TILs from the tumor microenvironment. This study also contributes to improving CRC subclassifications, not only in terms of microsatellite status, but also of the inflammasome pathway and the immune-related gene signature bridging innate and adaptive immunity. Altogether, our findings strongly suggest that targeting the inflammasome pathway could improve the anti-tumor immune response in subgroups of CRC.

## Figures and Tables

**Table 1:** Clinicopathological features of CRC patients and study design.

I. Clinicopathological Features of CRC Patients				
	Retrospective Cohort (*n* = 192)		Prospective Cohort (*n* = 96)	
	MSI (*n* = 43)	MSS (*n* = 149)	MSI (*n* = 22)	MSS (*n* = 74)
Age: mean (range)	74 (52–84)	68 (35–85)	74 (34–88)	68 (34–95)
Gender				
Men	15	101	6	42
Women	28	48	16	32
Tumor location				
Right	35	49	18	24
Transverse	5	12	0	0
Left	3	77	4	45
Rectum	0	11	0	5
pTNM stage (UICC)				
0	0	1	0	0
I	2	19	2	9
II	21	41	14	34
III	11	42	4	23
IV	9	46	2	8
Histological subtypes				
Adenocarcinoma NOS	22	129	13	61
Mucinous	9	13	7	12
Polymorph	2	4	0	0
Medullary	9	1	1	0
Signet ring cell	1	1	0	0
Serrated	0	1	1	1
II. Study design				
Retrospective cohort			Prospective cohort	
➢ Tissue microarray (TMA) ➢ Immunostaining: IL-18, Tbet and CD8 ➢ Semi-quantitative score of IL-18 expression in tumor cells ➢ Quantitative evaluation of TILs (Image J)			➢ Assessment of caspase-1 activity (FLICA assay) (*n* = 96) ➢ Immunostaining and quantification of Tbet, CD8, CD3, PD1-positive TILs (Imaris software) (*n* = 96) ➢ Ex vivo explant cultures of CRC and assessment of secreted cytokines (ELISA) (*n* = 96) ➢ Effect of rhIL-18 on TIL cell lines generated from CRC (*n* = 20) ➢ Transcriptomic analysis by qPCR (*n* = 16) or 3′RNA sequencing Illumina (*n* = 39)	

pTNM: Pathological Tumor Node Metastasis; UICC: Union for International Cancer Control; NOS: not otherwise specified; IHC: immunohistochemistry; rh: recombinant human.

## Data Availability

The data presented in this study are available on request from the corresponding author.

## Supplementary Materials

The following are available online at https://www.mdpi.com/2072-6694/13/2/189/s1. Figure S1: Hierarchical clustering after principal component analysis on CRC. Figure S2: CMS classification of CRC after 3′RNA sequencing using the CMS Classifier package. Table S1: Gene set enrichment analysis using ClueGo.
