# Supplementary figures and images for: The Caspase-1/IL-18 Axis of the Inflammasome in Tumor Cells: A Modulator of the Th1/Tc1 Response of Tumor-Infiltrating T Lymphocytes in Colorectal Cancer
Source: Cancers (Basel). 2021 Jan 7;13(2):189. doi: 10.3390/cancers13020189 (PMC7825767; doi:10.3390/cancers13020189)

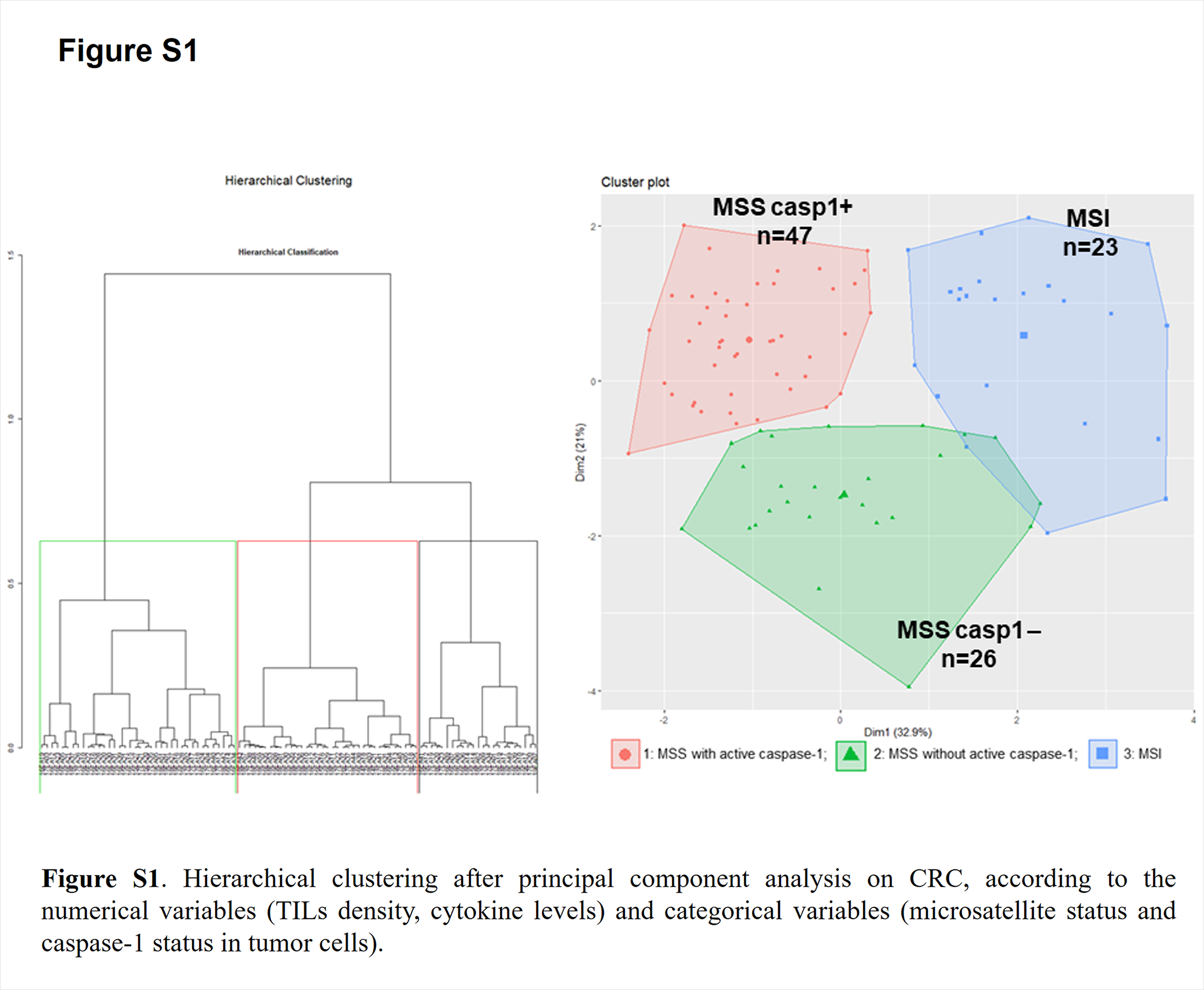

Supplement: Supplementary file 1 [file cancers-13-00189-s001.zip › Figures supp/Figure S1 colour.tif]

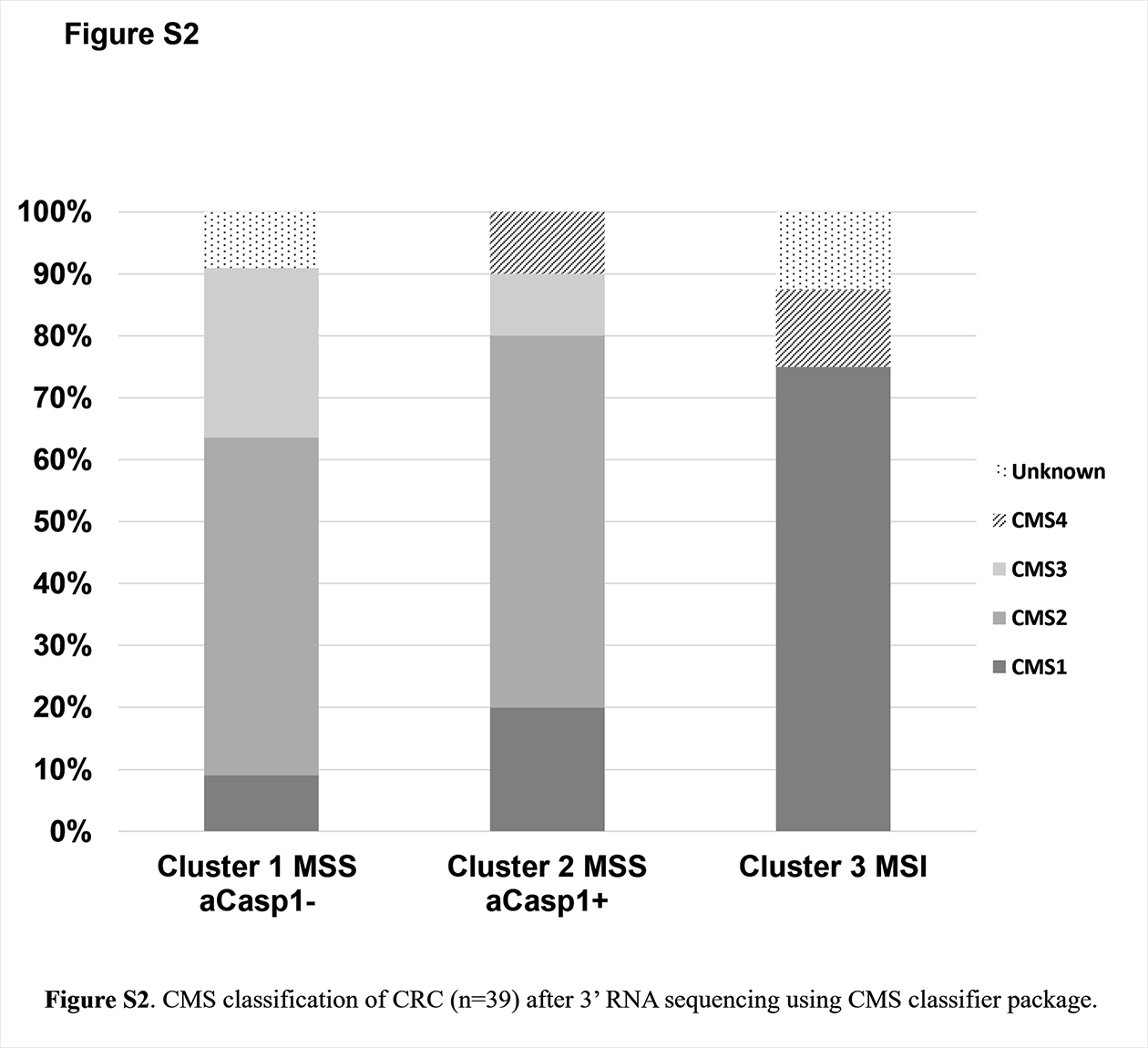

Supplement: Supplementary file 1 [file cancers-13-00189-s001.zip › Figures supp/Figure S2 mono.tif]

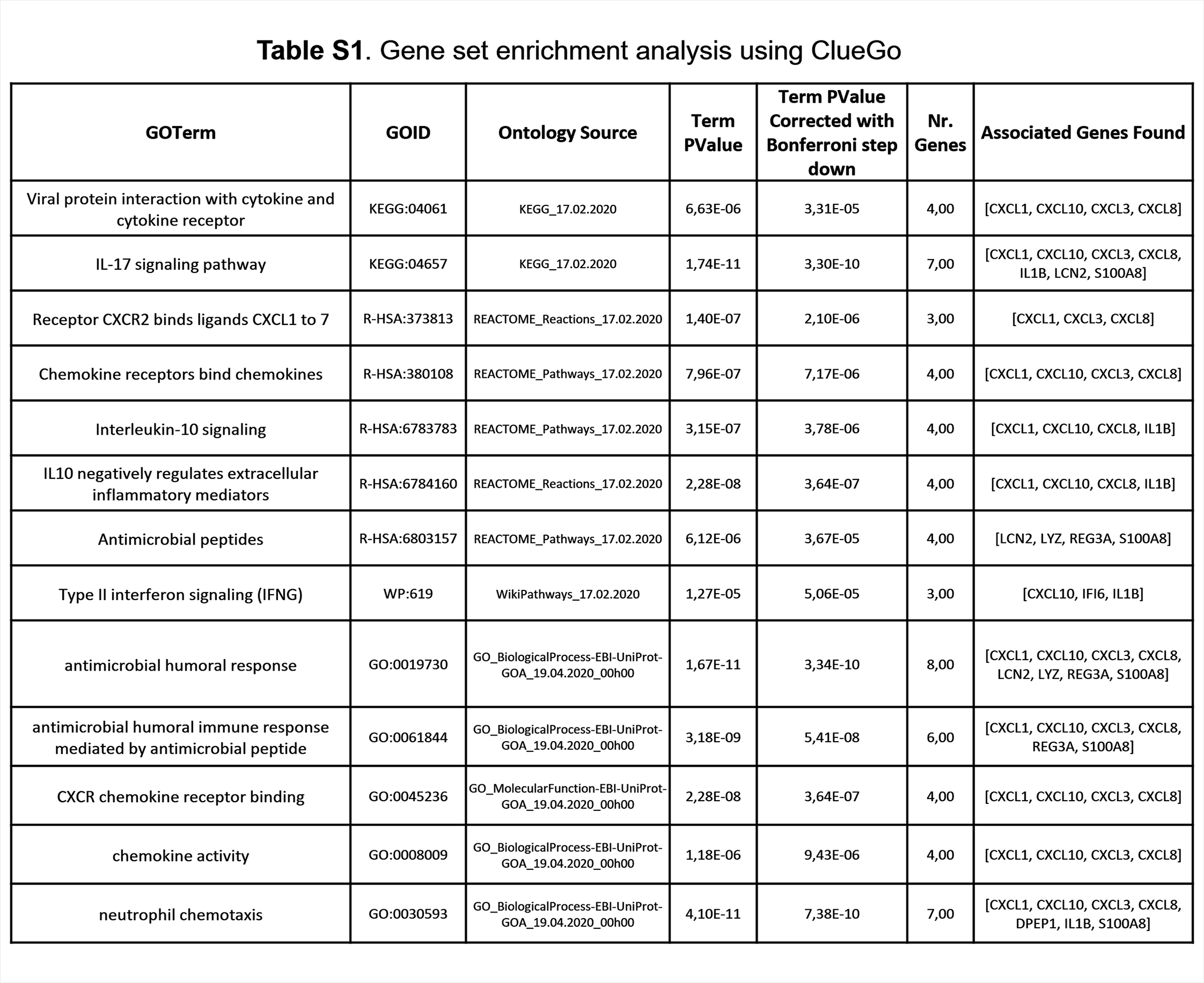

Supplement: Supplementary file 1 [file cancers-13-00189-s001.zip › Figures supp/Table S1.tif]
